# Analysis of the Reactivity of Z-2-Ar-1-EWG-1-Nitroethene Molecular Segment in the Hetero Diels–Alder Reaction: Experimental and MEDT Quantum Chemical Study

**DOI:** 10.3390/molecules30183768

**Published:** 2025-09-16

**Authors:** Przemysław Woliński, Agnieszka Kącka-Zych, Ewelina Wielgus, Rafał Dolot, Radomir Jasiński

**Affiliations:** 1 Cracow University of Technology, Faculty of Chemical Engineering and Technology, Department of Organic Chemistry and Technology, Warszawska 24, 31-155 Cracow, Poland; 2 Centre of Molecular and Macromolecular Studies, Polish Academy of Sciences, Sienkiewicza 112, 90-363 Łódź, Poland; ewelina.wielgus@cbmm.lodz.pl (E.W.); rafal.dolot@cbmm.lodz.pl (R.D.)

**Keywords:** hetero Diels–Alder reaction, cycloaddition, nitroalkene, reactivity, regioselectivity, mechanism

## Abstract

The relative reactivity of the nitrovinyl molecular segment characterized by the “cis” orientation of nitro group and the aryl ring was evaluated based on the experimental and Density Functional Theory quantum chemical data. It was found that, on the contrary to E-R-nitroethenes, the Z-2-Ar-1-EWG-1-nitroethene molecular segment is not planar. This fact reduces the possibility of the conjugation of π-electron systems, and as a consequence, decreases the global reactivity. Due to these conditions, the reaction of the model ethyl 4,β-dinitrocinnamate and 2-methylenecyclopentane is realized as a very difficult process; however, with full regioselectivity, it leads to the expected (4 + 2) hetero Diels–Alder cycloadduct. Bonding Evolution Theory studies show that the first new C4-C5 single bond is formed in Phase VIII by merging two *pseudoradical* centers. In turn, the second C6-O1 single bond is formed in last phase of the reaction, by the depopulation of V(C6), V(O1) and V’(O1) monosynaptic basins. According to this, the title reaction was classified as a process carried out according to a “*one-step two-stage*” mechanism.

## 1. Introduction

The nitrogroup plays an important role as a key molecular segment within many biologically active compounds [1,2,3,4,5,6,7]. The presence of the nitrogroup in organic molecules additionally offers a wide range of potential paths for the further functionalization of amines [8], imines [9,10], oximes [11], nitrile N-oxides [12], six-membered nitronates [13] and other functionalized structures [8]. Within different types of nitrocompounds, the exhibiting of conjugated nitroalkenes (CNAs) is especially important, due to the electrophilically activated vinyl moiety. Additional activation by the geminal electron-withdrawing group (EWG) stimulates the high reactivity of this group of organic molecules. For example, these type of activated compounds react very rapidly via a (3 + 2) cycloaddition (32CA) scheme with nitrones [14], diazocompounds [15], nitrile N-oxides [16] and azomethine ylides [17]. Next, the electrophilic acceleration is clearly visible in the case of analogous (4 + 2) cycloaddition (42CA) processes.

**Scheme 1:**
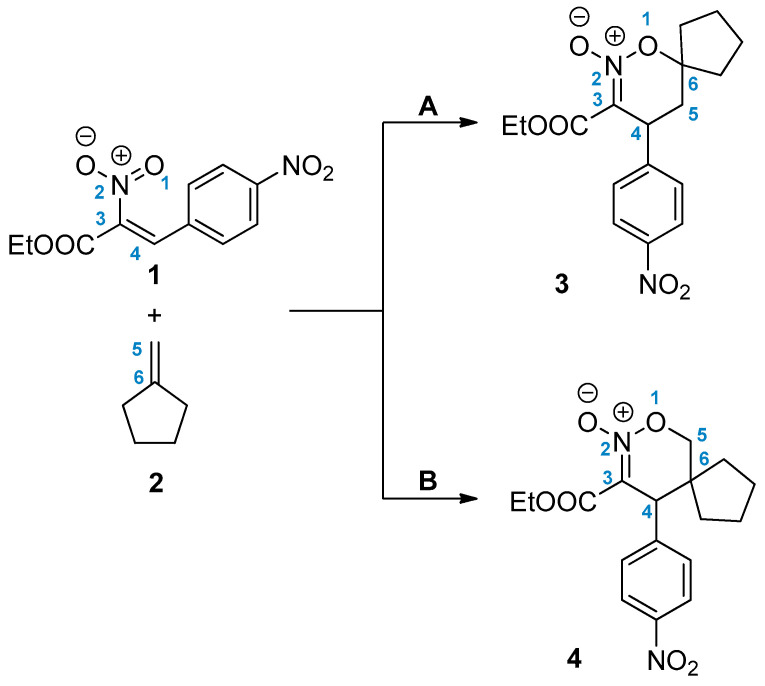
Theoretically possible, regioisomeric paths of the HDA reaction between ethyl 4,β-dinitrocinnamate (**1**) and methylenecyclopentane (**2**).

In general, the vast majority of conjugated nitroalkenes exist in stable forms characterized by the trans orientation of the nitro group and the substituent at the 2-position. For example, 2-alkyl-1-nitroethenes [18,19], 2-CX_3_-1-nitroethenes [20,21,22], 2-EWG-nitroethenes [18,23], 2-aryl-1-halo-1-nitroethenes [24,25], and 2-aryl-1-cyano-1-nitroethenes [26,27] can be prepared only in stable E-configuration. Analogs characterized by an inverse geometric configuration are known but are much rarer [28,29]. The rare example of a CNA characterized by stable Z-configuration is ethyl 4,β-dinitrocinnamate (DNC) (**1**), which was described for the first time in 1981 [30]. At present, the physical and chemical properties of this compound are, however, not satisfactorily known. Our work includes a new, important contribution in this area. Within our research, we decided to shed light on the relative reactivity of the DNC in the hetero Diels–Alder (HDA) reaction; this is in comparison to recent similar studies on the reactivity of a series of E-2-aryl-1-cyano-1-nitroethenes [31,32,33,34]. As a model partner for the DNC, we selected sterically crowded 2-methylenecyclopentane (**2**), which was tested earlier in analogous reactions with E-R-nitroethenes [32]. The HDA reaction between mentioned addents can be theoretically realized according to two competitive, regioisomeric paths, leading to regioisomeric oxazine N-oxides **3** and **4** (Scheme 1).

In the framework of our research we decided to (i) analyze the electronic structures of reaction components based on the Conceptual Density Functional Theory (CDFT [35]) approach, and an Electron Localization Function (ELF [36]) study; (ii) search for optimized reaction conditions and a methodology for analyzing postreaction mixtures, as well as for isolating reaction product(s); (iii) identify reaction products and determine reaction regioselectivity; (iv) explain the reaction course based on the full exploration of reaction profiles for both theoretically possible regioisomeric paths; and (v) interpret electron density reorganization on the reaction path in practice, based on Bonding Evolution Theory (BET [37]). We hope that this type of experimental and theoretical study offers a comprehensive view on the problem of the global and local reactivity of the title nitrovinyl molecular segment in comparison with E-2-Ar-1-EWG-nitroethenes.

## 2. Results and Discussion

**Figure 1:**
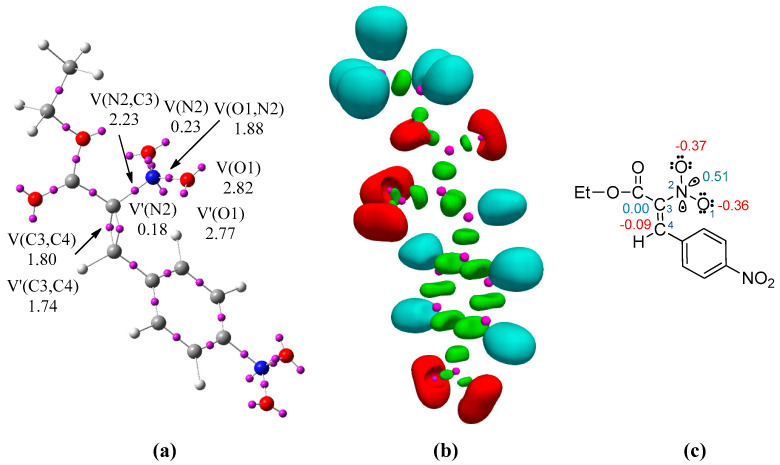
(**a**) ELF basin attractor positions, together with the most representative valence basin populations; (**b**) ELF localization domains of ethyl 4,β-dinitrocinnamate (**1**), represented at an isosurface value of ELF = 0.80; (**c**) Lewis-like structure of **1** together with the natural atomic charges, obtained through an NPA. Negative charges are colored in red, positive charges are colored in green and neutral in blue. Natural atomic charges are given in average number of electrons, e.

In order to characterize the electronic structure of DNC (**1**), a quantum chemical analysis of Becke and Edgecombe’s ELF [36] was conducted. Figure 1 shows the most important valence basin populations and ELF localization domains.

ELF topological analysis of **1**, in the most important region, shows the presence of two V(C3,C4) and V’(C3,C4) disynaptic basins integrating 1.80 e and 1.74 e, respectively. These disynaptic basins are associated with the presence of a double bond in structure **1**. In **1**, we also notice the presence of two disynaptic basins, V(N2,C3) and V(O1,N2), integrating 2.23 e and 1.88 e, respectively. The presence of these basins indicates the occurrence of two N2-C3 and O1-N2 single bonds. On the nitrogen N2 and oxide O3 atoms, we observe the occurrence of monosynaptic basins related to the presence of free electron pairs (Figure 1). The electronic structure of the methylenecyclopentane (**2**) was detailed and described in our previous work [32].

Next, the charge distribution was analyzed through Natural Population Analysis (NPA) [38]. Natural atomic charges of the most relevant atoms are shown, together with the proposed ELF-based Lewis-like structures given in Figure 1c. Based on the NPA of **1**, we can distinguish that the C4 and O1 atoms accumulate negative charges of −0.08 e and −0.36 e, respectively. In turn, the N2 atom has a positive charge of 0.51 e. It is worth nothing that the carbon C3 is neutral.

Next, we also decided to check the global and local electronic properties of ethyl 4,β-dinitrocinnamate (1) (Table 1). It can be seen that the electrophilicity [39] ω and nucleophilicity [40] N indices of **1** are 3.32 and 1.72 eV, respectively, classifying it as a strong electrophile and moderate nucleophile within the electrophilicity [41] and nucleophilicity [42] scales. From these values, it is expected that **1** will react as a strong electrophile. Analysis of the electrophilic P_k_^+^ of ethyl 4,β-dinitrocinnamate (**1**) indicates that the C4 carbon atom is the most electrophilic center in this structure (P_C4_^+^ = 0.24). For the C4 carbon atom, the value of local electrophilicity is ω_C4_ = 0.81 eV.

**Figure 2:**
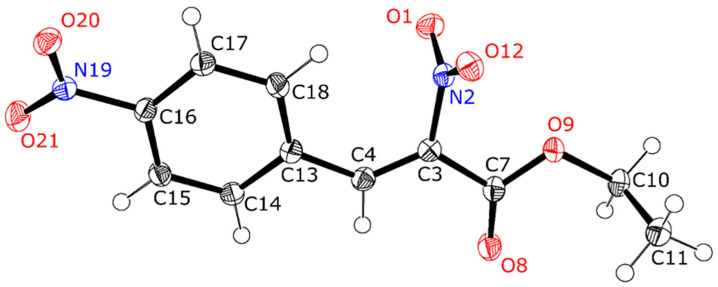
The molecular structure of compound **1** (DNC). Ellipsoids are shown at 50% probability.

For the preparation of CNAs, several strategies are employed. Most universal approaches are based on the dehydratation of 2-nitroalcohols or thermal decomposition of their esters [43,44,45]. These type of protocols have, however, failed in the synthesis of the DNC. We therefore decided to prepare the target compound via condensation of the ethyl nitroacetate with N-n-propyl-C-(4-nitrophenyl)imine. We confirmed its geometrical isomerism based on the HRMS experiments and X-ray analysis. The high-resolution mass spectrum was obtained using the atmospheric pressure chemical ionization technique. It was found that, in negative mode, a peak at *m*/*z* 266.0540 was observed, which corresponded with the proposed C_11_H_10_N_2_O_6_ molecular formula. It is worth noting that DNC forms a radical anion [M]^−^ in the APCI(−)-MS, which is characteristic of nitro compounds. These radical anions undergo further fragmentation by losing neutral molecule NO_2_ (*m*/*z* 219.0532). Unfortunately, assignment geometrical isomerism is not possible based on mass spectrometry analysis. The X-ray diffraction experiment performed on a single crystal of DNC gave good crystallographic data with a high resolution (Table 2). The obtained model of the structure confirmed that the compound used is a Z-isomer. Note that the aliphatic nitro group is not coplanar with the C=C bond (Figure 2). This conclusion is consistent with the X-ray analyses performed for this compound 43 years ago [46].

Based on the fully defined DNC **1**, we initiated the tests to search for acceptable reaction conditions. We evaluated several samples, using different temperatures, reaction times, solvents and molar reagent ratios. These tests exhibited the evidently low reactivity of the tested nitroalkene. Applying the cycloaddition protocol analogously, as in similar reactions with the participation of E-2-aryl-1-cyano-1-nitroethenes [32,33], was not successful. We found that the cycloaddition process can be realized in toluene solution at 80 °C. After 72 h, the consumption of the nitroalkene was finished. The analysis of the crude postreaction mixture exhibited that, in the course of the reaction, only one cycloaddition product is formed. This product was isolated using column chromatography (SiO_2_; AcOEt/Petroleum Ether 30/70) and identified on the basis of spectral analysis. In particular, within the IR spectrum, signals characteristic of the nitro group at the phenyl ring were detected. At the same time, the nitronate characteristic band at 1596 cm^−1^ was localized instead of the signal derived from the aliphatic nitro group. In the ^1^H NMR spectrum, signals characteristic of the geminal aliphatic protons at the sp^3^ carbon atom were identified. In the relatively strong field, the signal of protons of the cyclopentane skeleton were also localized. Next, in a relatively weaker field, we detected signals of aromatic protons. For full characterization of this compound, the ^13^C NMR spectrum was also registered. Furthermore, important information regarding the structure of isolated compound aids in the detailed mass spectrometry analysis. The high-resolution mass spectra recorded using the electrospray ionization technique give peaks corresponding to protonated molecular ions in positive mode and deprotonated molecular ions in negative mode. The elemental composition of the peak at *m*/*z* 349.1405 in positive mode and the peak at *m*/*z* 347.1241 in negative mode confirmed the molecular formula C_17_H_20_N_2_O_6_. Analysis of fragmentation ions showed that one of the basic peaks in the negative ESI MS spectrum at *m*/*z* 263.0668 arises as a result of the elimination of C_5_H_8_O. In the case of isomer **3**, such a fragmentation process may occur due to the cleavage of O1-N2 and C5-C6 bonds. It is difficult to propose a fragmentation mechanism that leads to the formation of such an ion for isomer **4**, allowing us to conclude that the cycloaddition reaction results in the formation of regioisomer **3**.

**Figure 3:**
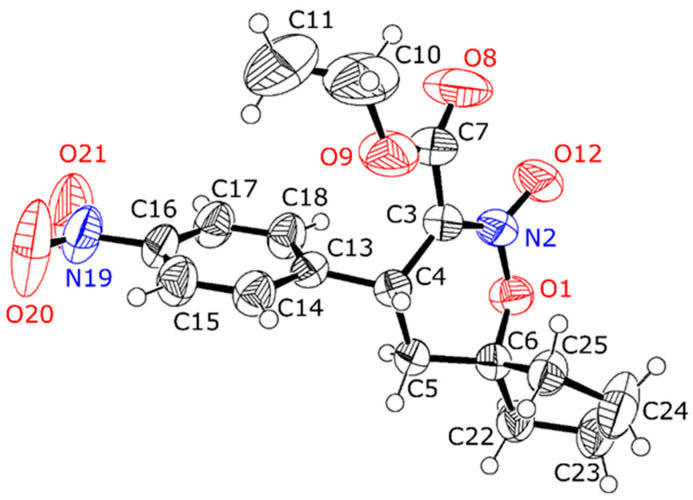
The molecular structure of compound **3**. Ellipsoids are shown at 50% probability.

Finally, an X-ray analysis of the crystals obtained from the crystallization of the reaction product clearly confirmed that the compound analyzed was regioisomer **3** (Figure 3). Note that the crystals of compound **3** cracked when exposed to 100 K, both with and without the use of cryoprotectant (mineral oil). Measurements at room temperature gave unsatisfactory results due to the high thermal vibration factors of the atoms. The solution was to carry out a diffraction experiment at 273 K (see Table 2).

**Figure 4:**
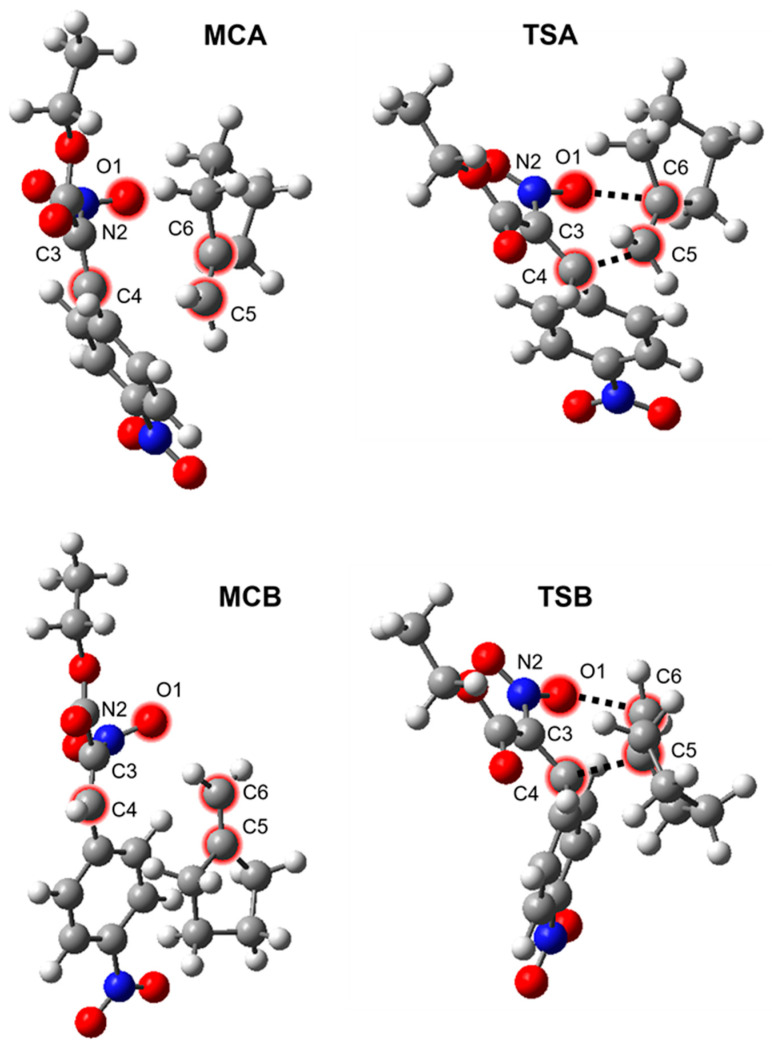
View of critical structures for the HDA reaction between ethyl 4,β-dinitrocinnamate (DNC) (**1**) and methylenecyclopentane (**2**) in the toluene solution in light of the ωB97xd/6-311+G(d) (PCM) calculations.

In the next step of the research, we evaluated both competitive reaction channels on the basis of the results of the ωB97xd/6-311+G(d) (PCM) calculations. It was found that the nature of both profiles in the toluene solution is similar and that they include two critical points along the reaction coordinates. These critical points are connected to the existence of respective pre-reaction complexes (**MCA** for the path **A**, and **MCB** for the path **B,** respectively), as well as with the existence of respective transition states (**TSA** for the path **A**, and **TSB** for the path **B,** respectively). It should be underlined at this point that the quantitative description of profiles is substantially different (Table 3 and Figure 4). The interactions between reagents in the initial reaction phase, independent of the reaction channel, lead to the formation of the respective pre-reaction complexes. This process is associated with the reduction in the enthalpy of the reaction system by a few kcal/mol, and is realized as barrier-less. It should be mentioned that, during the formation of MCs, the entropy of the reaction system is dramatically reduced. As a consequence, the Gibbs free energies of the formation of MCs are positive. This factor excludes the possibility of the existence of pre-reaction complexes as relatively stable intermediates. Their formation is a consequence of the tendency to adopt addends in the orientation, which is attractive from the point of view of the nature of coulombic interactions. Subsequently, all reaction centers arrange the orientation, which determines the further reaction regioselectivity. The key interatomic distances exist, however, beyond the range typical for new sigma C-C and C-O bonds in transition states [17,47,48]. The electron density is also not transferred between reaction substructures at this stage (GEDT = 0.00 e). Therefore, localized intermediates should be classified as orientation [49,50,51], but not charge transfer, complexes [14,52,53].

**Figure 5:**
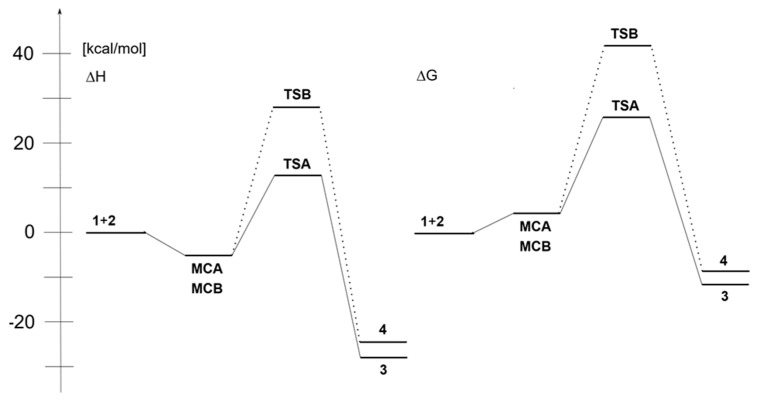
Energetical profiles for the HDA reaction between ethyl 4,β-dinitrocinnamate (DNC) (**1**) and methylenecyclopentane (**2**) in the toluene solution in light of the ωB97xd/6-311+G(d) (PCM) calculations.

The further conversion of pre-reaction complexes is associated with the increasing energy of the reaction system and always leads to the area of the respective transition states (**TSA** for the path **A**, and **TSB** for the path **B,** respectively). The activation barriers on considered paths are, however, substantially different. In particular, for path **A,** the enthalpy of the activation does not exceed 12 kcal/mol. For comparison, on competitive path **B**, the activation enthalpy is equal to almost 28 kcal/mol. So, in the considered cycloaddition, path **B** should be treated as forbidden from a kinetic point of view (Figure 5). Both optimized TSs exhibit a two-planar structure nature, which is typical for DA reactions [54,55,56]. The synchronicities of the analyzed TSs are substantially different (Figure 4). In particular, the difference between key distances within **TSAs** are higher than 0.9 A, whereas the analogous parameter for the **TSB** is equal to about 0.4 A. At the same time, there is a great electron density flux from the substructure of the 2-methylenecyclopentane (**2**) to the substructure of the nitroalkene (GEDT > 0.4 e) (Table 4). As a consequence, the considered process should be treated as evidently polar. All attempts to localize zwitterionic intermediates along the reaction coordinate were not successful. Next, IRC calculations directly connect both optimized TSs with valleys of respective MCs and cycloadducts (**3** and **4** for paths **A** and **B**, respectively).

At the same time, we also performed an analogous study on isomeric E-DNC. It was found that the Gibbs free energy of the activation for path **A** was 1 kcal/mol lower than in the case of the analogous path in the title reaction. The second theoretically possible regioisomeric path **B** should be treated as forbidden from a kinetic point of view (ΔG ≠ ~40 kcal/mol). So, in the reaction with the planar orientation of the nitro group, the vinyl moiety (E analog of the DNC), the reactivity of the CNA is relatively better, while that in the case of the DNC characterized by Z geometry. The reactivity of the Z analog of the DNC can be theoretically higher; unfortunately, the carboalkoxy group adopts, in this case, non-planar orientation to the vinyl moiety.

**Scheme 2:**
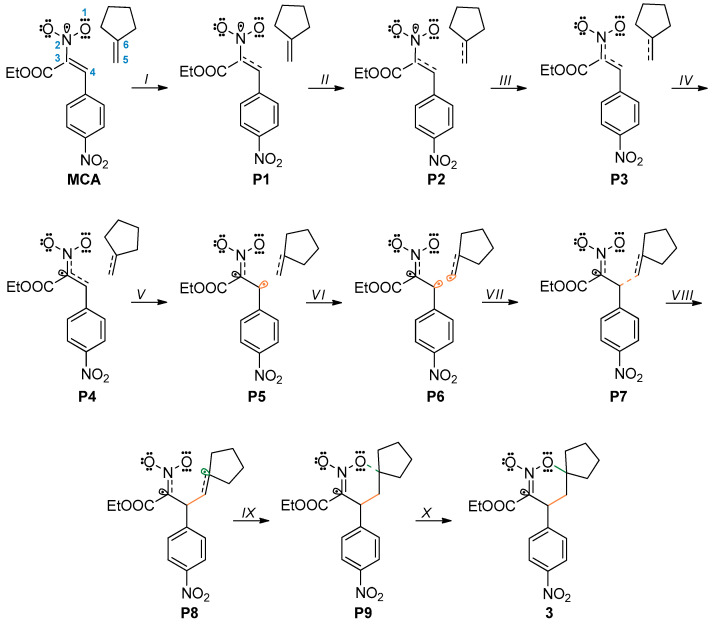
A simplified representation of the molecular mechanism of the HDA reaction between ethyl 4,β-dinitrocinnamate (DNC) (**1**) and methylenecyclopentane (**2**) by Lewis-like structures arising from topological ELF analysis along the reaction path.

In order to understand the bonding changes along the HDA reaction between ethyl 4,β-dinitrocinnamate (DNC) (**1**) and methylenecyclopentane (**2**), BET analysis was carried out. Populations of the most significant valence basins of selected structures of the IRC are collected in Table 5 and the proposed molecular mechanism represented by Lewis-like structures resulting from the ELF topology is shown in Scheme 2.

**Figure 6:**
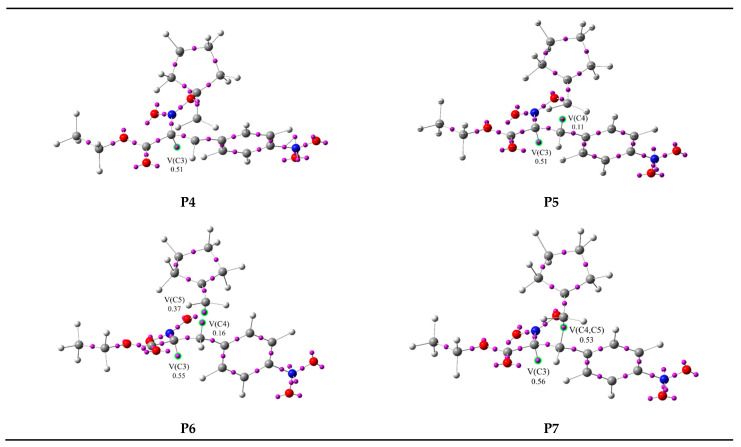

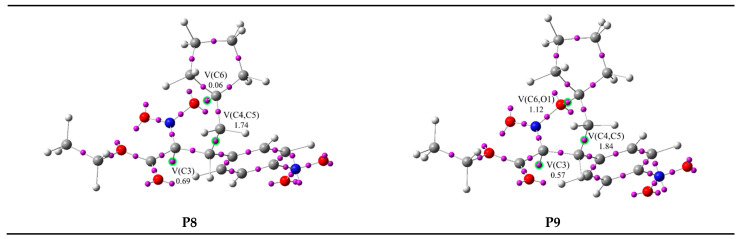
The most significant attractor positions of the ELF valence basins of the **P4**–**P9 structures** participating in the C4-C5 and C6-O1 single-bond formation, participating in the HDA reaction between ethyl 4,β-dinitrocinnamate (DNC) (**1**) and methylenecyclopentane (**2**). The electron populations, in average number of electrons, are given in e.

In the analyzed mechanism, we can distinguish ten different topological phases (Table 5). Along *Phase I*, we observed only slight changes in relation to separated reagents. The topological features of the ELF of the corresponding molecular complex, **MCA**, which is the first point of the IRC, are very similar to those of the isolated reagents. We can only notice a slight change due to the disappearance of the V’(N2) monosynaptic basin located on atom N2, integrating 0.18e in **1**. Based on this, we notice an increase in the population of the V(N2,C3) disynaptic and V(N2) monosynaptic basins (Table 5). *Phase II* begins at the structure **P1**. Along this phase, the two V(C3,C4) and V’(C3,C4) disynaptic basins present in *Phase I* have merged into one V(C3,C4) disynaptic basin, integrating 3.47 e. This change is associated with the breaking of the C3-C4 double bond in **1**. *Phase III* starts at the structure **P2**. This phase is related to the rupture of the C5-C6 double bond in molecule **2**. We observed the disappearance of one V’(C5,C6) disynaptic basin present in a previous phase and the formation of a new V(C5, C6) disynaptic basin with a population of 3.29 e. In the next phase, *Phase IV*, we observe the disappearance of the V(N3) monosynaptic basin associated with the disappearance of the lone electron pair on the N3 atom. As a result, we see an increase in the population of the V(N2,C3) disynaptic basin, integrating 2.56 e (Table 5). *Phase V* starts at the structure **P4**. This phase begins with the creation of a C3 *pseudoradical* center at the ethyl 4,β-dinitrocinnamate (**1**) moiety, integrating 0.51 e (see V(C3) in Table 5 and Figure 6). In the next phases, *Phases VI* and *VII* (which start at **P5** and **P6**, respectively), we observed the creation of two *pseudoradical* centers at C4 and C5 carbon atoms. The formation of these *pseudoradical* centers is mainly prompted by the depopulation of the C5-C6 and C3-C4 bonding regions (Figure 6 and Table 5). *Phase VIII* starts at **P7**. The most relevant topological change is evidenced here: the two C4 and C5 *pseudoradical* centers merged into new C4-C5 bonding regions with an initial population of 0.53 e (see V(C4,C5) in Table 5 and Figure 6). This phase is related to the formation of a new C4-C5 bond. *Phase IX* starts at the structure **P8**. This phase begins with the creation of a C6 *pseudoradical* center at the methylenecyclopentane (**2**) moiety, integrating 0.06 e (see V(C6) in Table 5 and Figure 6). The next phase, *Phase X*, is associated with the second important change occurring during the HDA reaction between ethyl 4,β-dinitrocinnamate (DNC) (**1**) and methylenecyclopentane (**2**). In this phase, we observed the formation of a new V(C6,O1) disynaptic basin by merging the V(C6) monosynaptic basin and depopulation of V(O1) and V’(O1) monosynaptic basins located on O1 atom. These changes are related to the formation of a second C6-O1 single bond. Finally, at compound **3**, the electron population is relaxed: the C4-C5 and C6-O1 bonding regions integrate 1.84 e and 1.43 e. The N2-C3 bonding region reaches incremented and symmetric populations of 3.60 e, acquiring the expected population for double bonds (Table 5).

From the BET analysis of the HDA reaction between ethyl 4,β-dinitrocinnamate (DNC) (**1**) and methylenecyclopentane (**2**), we can state that (i) this HDA reaction proceeds along ten different phases, related to the change the electron density along the reaction path (Scheme 2). (ii) At the beginning of the reaction, we observe the breaking of two C3-C4 and C5-C6 double bonds. (iii) The first new C4-C5 single bond is formed in *Phase VIII* by merging two C4 and C5 *pseudoradical* centers into a new C4-C5 bonding region with an initial population of 0.53 e. (iv) The second C6-O1 single bond is formed in the last phase, by merging the V(C6) monosynaptic basin and the depopulation of V(O1) and V’(O1) monosynaptic basins. (v) Based on the BET analysis, we can conclude that the title reactions proceed according to the “*one-step two-stage*” mechanism (Scheme 2).

## 3. Materials and Methods

### 3.1. Analytical Techniques

HPLC analyses were carried out using a Knauer device with a UV VIS detector (LiChrospher 18-RP 10 µm column, eluent: 80% methanol). M.p. values were measured on the Boetius apparatus and were uncorrected. IR spectra were derived from the FTS Nicolet IS 10 spectrophotometer. ^1^H NMR and ^13^C NMR spectra were recorded on a Bruker Avance III 500 spectrometer, and are reported in ppm using deuterated solvent as an internal standard (CDCl_3_ at 7.26 ppm). High-resolution mass spectrometry (HRMS) measurements were carried out on a Synapt G2-S*i* mass spectrometer (Waters) equipped with an electrospray (ESI) and atmospheric pressure chemical ionization (APCI) source and a quadrupole time-of-flight mass analyzer. Spectra were recorded in positive or negative ion modes over a mass range of *m*/*z* 50–1200 in centroid mode with an acquisition time of 1 min. Mass accuracy was maintained using leucine enkephalin as an external reference (LockSpray™), which provided ions at *m*/*z* 556.2771 ([M + H]^+^) in positive mode and *m*/*z* 554.2615 ([M − H]^−^) in negative mode. Data processing was performed using MassLynx 4.2 software (Waters).

### 3.2. X-Ray Crystal Structure Determination

Single-crystal X-ray diffraction data were recorded using a Rigaku XtaLAB Synergy-S diffractometer with a CuKα source (*λ* = 1.5418 Å) and a HyPix–6000HE detector. Crystals of compounds **1** and **3**, grown from a 1:1 (*v*/*v*) toluene–cyclohexane mixture, were mounted on cryo-loops in mineral oil. Compound **1** was flash-cooled to 100 K in a N_2_ stream, while compound **3** was cooled to 273 K due to its instability at lower temperatures. Data acquisition strategies were created in CrysAlisPro (Rigaku, ver. V1.171.43.122a, 2024). Structures were solved with SHELXT (2018/2) using intrinsic phasing [57], refined with SHELXL (2018/3) [57] and visualized in Olex2 [58]. Non-hydrogen atoms were refined anisotropically; hydrogen atoms were placed geometrically and refined with a riding model. The final structures were validated with CheckCIF (http://checkcif.iucr.org, accessed on 1 September 2025) and deposited at the CCDC under accession numbers 2471984 and 2471985.

### 3.3. Synthesis of Reaction Components

The methylenecyclopentane (**2**) was prepared according to a multi-step procedure described in the literature [30,32]. For the preparation of the 4,β-dinitrocinnamate (DNC) (**1**), the procedure employed and improved upon in our laboratory was as follows:

#### 3.3.1. Stage 1—Preparation of the N-n-propyl-c-(4-nitrophenyl)imine

In a round-bottomed flask, equipped with a magnetic stirrer, 11.25 g (0.074 mol) of 4-nitrobenzaldehyde, 4.43 g (6.2 cm^3^, 0.075 mol) of n-propylamine, 4.00 g of anhydrous magnesium sulfate (VI) and 50 cm^3^ of dichloromethane were placed. The mixture was stirred for 24 h, after which the precipitate was filtered on a sintered funnel and washed with two 5 cm^3^ portions of the dichloromethane; the obtained filtrate was evaporated on an evaporator. The obtained precipitate was mixed with 20 cm^3^ of the petroleum ether (b.p.: 40–60 °C), filtered and dried. We obtained 14.30 g (99%) of N-n-propyl-C-(4-nitrophenyl)imine. M.p.: 49–51 °C ([59]: 53 °C).

#### 3.3.2. Stage 2—Preparation of the Ethyl 4,β-Dinitrocinnamate (DNC)

In a round-bottomed flask equipped with a thermometer and a magnetic stirrer, 7.09 g (0.0369 mol) of N-n-propyl-C-(4-nitrophenyl)imine and 5.4 g (4.5 cm^3^, 0.0406mol) were placed, along with ethyl nitroacetate (a commercially available substance was used) and 7.00 cm^3^ of acetic anhydride. The mixture was heated to 60 °C and stirred for 1 h, after which a yellow precipitate was precipitated, which was filtered on a sintered funnel and washed, first with three 5 cm^3^ portions of warm water and then with two 5 cm^3^ portions of petroleum ether (b.p.: 40–60 °C). After drying, the crude product was crystallized from a mixture of toluene and cyclohexane. We obtained 5.47 g (55.7%) of ethyl 4,β-dinitrocinnamate. M.p.: 108.1–110.5 °C ([60]: 109–111 °C). HRMS (-APCI): *m*/*z* calculated for C_11_H_10_N_2_O_6_: 266.0539 [M]^−^; measured 266.0540.

### 3.4. Preparation of 3-Carboethoxy-4-nitrophenyl-6,6-spirocyclopentyl-1,2-oxazine N-Oxide (**3**)

The mixture of 0.5324 g (0.002 mol) of the ethyl 4,β-dinitrocinnamate (DNC) (**1**) and methylenecyclopentane (**2**) 0.1971 g (0.0024 mol) in 5 cm^3^ of dry toluene was heated at 80 °C for 72 h. The postreaction mixture was analyzed by HPLC and the reaction product was isolated using column chromatography (SiO_2_; AcOEt/Petroleum Ether 30/70). The crude cycloadduct was recrystallized from the methanol and identified based on spectral characteristics and Rtg analysis. M.p.: 121–123 °C (MeOH). Colorless crystals. TLC: Rf 0.17 (SiO_2_; AcOEt/Petroleum Ether 30/70). ^1^H NMR: (500 MHz, (CD_3_)_2_SO, ppm) δ 8.16 (d, *J* = 8.62 Hz 2H), 7.51 (d, *J* = 8.62 Hz, 2H), 4.50 (dd, *J* = 10.23, 8.03 Hz, 1H), 3.97–3.87 (m, 2H), 2.36 (dd, *J* = 13.65, 8.03 Hz, 1H), 2.02 (dd, *J* = 13.65, 10.23 Hz, 1H), 1.94–1.89 (m, 1H), 1.82–1.76 (m, 2H), 1.73–1.65 (m, 4H), 1.60–1.57 (m, 1H), 0.85 (t, *J* = 7.12 Hz, 3H) ppm. ^13^C NMR: (125 MHz, (CD_3_)_2_SO; ppm) δ 160.8 (C), 150.1 (C), 147.0 (C), 129.2 (CH), 124.5 (CH), 116.9 (C), 95.0 (C), 61.7 (CH_2_), 40.5 (CH), 38.7 (CH_2_), 37.8 (CH_2_), 34.2 (CH_2_), 24.6 (CH_2_), 23.9 (CH_2_), 14.0 (CH_3_) ppm. FT IR (ATR; cm^−1^): 3077, 2966, 2934, 2873, 2453, 2285, 1801, 1725, 1690, 1606, 1596, 1562, 1518, 1493, 1463, 1447, 1433, 1384, 1364, 1344, 1309, 1234, 1212, 1175, 1158, 1132, 1106, 1044, 1015, 998, 952, 912, 895, 871, 853, 833, 801, 778, 761, 750, 694, 674, 640, 623, 562, 540, 501, 454, 437, 420, 406. HRMS (+ ESI): *m*/*z* calculated for C_17_H_21_N_2_O_6_: 349.1394 [M + H]^+^; measured 349.1405; HRMS (– ESI): *m*/*z* calculated for C_17_H_19_N_2_O_6_: 347.1243 [M – H]^−^ measured 347.1241.

### 3.5. Quantum Chemical Calculations

Analyses of molecular mechanisms were performed on the basis of the quantum chemical DFT calculations. For this purpose, the ωB97xd/6-311+G(d) (PCM) level of theory from the Gaussian software package [61] was used. This was the same functional we used previously for the exploration of the regio- and steroselectivity and the molecular mechanism of many different types of cycloaddition processes [22,32,54,55]. Within these studies, we received satisfactory correlations between DFT predictions and the experimental results. All localized and optimized stationary points were examined via vibrational analysis. It was found that starting molecules, intermediates (pre-reaction, **MCA** and **MCB** complexes) and products had positive Hessian matrices. On the other hand, all transition states (**TSA** and **TSB**) showed only one negative eigenvalue in their Hessian matrices. The calculations were performed for T = 297 K.

Intrinsic reaction coordinate (IRC) calculations were implemented for the verification of all optimized transition states. The presence of the solvent (toluene) in the reaction environment was included using the IEFPCM solvation model. Global electron density transfer (GEDT) [62] within transition states (where it corresponds specifically to the nitroalkene fragment) was calculated according to the following formula:

GEDT = −ΣqA(1)

where qA is the net charge and the sum is taken over all the atoms of nitroalkene substructure.

Global electronic properties of reaction components were calculated using equations recommended by Parr and Domingo [39,40]. According to Domingo’s suggestions, for this purpose, the ωB97xd/6-311G(d) level of theory was used. All molecules were fully optimized. Next, the electronic chemical potentials (µ) and chemical hardness (η) were evaluated in terms of one-electron energies of FMO (E_HOMO_ and E_LUMO_) using the following equations:

μ ≈ (E_HOMO_ + E_LUMO_)/2  η ≈ E_LUMO_ − E_HOMO_ (2)

Next, the values of µ and η were used for the calculation of global electrophilicity (ω) [41] according to the following formula:

ω = μ^2^/2η (3)

Subsequently, global nucleophilicity (N) [42] can be expressed in terms of the following equation:

N = E_HOMO_ − E_HOMO (tetracyanoethene)_ (4)

The local electrophilicity (ω*_k_*) condensed to atom k was calculated by projecting the index ω onto any reaction center *k* in the molecule using Parr functions P*_k_*^+^ [42]:

ω*_k_* = P^+^*_k_*·ω (5)

The same level of theory was used within ELF analysis. ELF [36] studies were performed with the TopMod package [63], considering the standard cubical grid with a step size of 0.1 Bohr. The bonding changes along corresponding reactions were analyzed, according to BET [39], by performing a topological analysis of the ELF for 600 nuclear configurations for reactions leading to product **3**. The ELF molecular geometries and basin attractor positions were visualized using the GaussView program [64]. ELF localization domains were represented by using Paraview software at an isovalue of 0.80 a.u [65,66].

## 4. Conclusions

This comprehensive, experimental and DFT quantum chemical study confirms, without any doubts, that the reactivity of the Z-(ethyl 4,β-dinitrocinnamate) is dramatically different than seemingly similar E-2-aryl-1-cyano-1-nitroethenes. We found that the reaction of the title nitroalkene with methylenecyclopentane is realized under very difficult conditions, in contrast to the analogous, rapid reactions involving *E*-2-aryl-1-cyano-1-nitroethenes. This phenomenon can be explained on the basis of the analysis of geometrical parameters of electrophilic agents and their electronic structures derived via ELF studies. Detailed BET analysis of the HDA reaction between ethyl 4,β-dinitrocinnamate (**1**) and methylenecyclopentane (**2**) indicates that we can distinguish ten different phases in this process. The analysis allowed us to classify the title reactions as processes proceeding according to the “*one-step, two-stage*” mechanism.

## Figures and Tables

**Table 1:** Global and local electronic properties of ethyl 4,β-dinitrocinnamate (DNC) (**1**).

	Global Properties				Local Properties		
	µ [eV]	η [eV]	ω [eV]	N [eV]	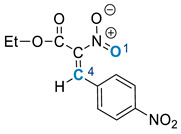 **1**		
						P_k_^+^	*ω*_k_
**1**	5.29	4.22	3.32	1.72	**C4**	0.24	0.81
					**O1**	0.05	0.17

**Table 2:** Crystal structure, data collection, and refinement parameters for compound **1** and compound **3** crystals.

Compound	Compound 1	Compound 3
**Crystal data**		
CCDC	2471984	2471985
Chemical formula	C_11_H_10_N_2_O_6_	C_17_H_20_N_2_O_6_
Formula weight	266.21	348.35
Crystal system	Triclinic	Triclinic
Space group	*P*1	*P*1
Temperature (K)	99.95(13)	272.99(14)
*a* [Å]	7.6679(2)	7.2551(1)
*b* [Å]	7.7157(1)	9.2996(2)
*c* [Å]	10.9464(2)	13.9423(2)
*α* [°]	82.178(2)	71.538(1)
*β* [°]	76.778(2)	85.035(1)
*γ* [°]	68.692(2)	77.182(1)
*V* [Å^3^]	586.36(2)	869.90(3)
Z	2	2
Z′	1	1
d_calc_ [g/cm^3^]	1.508	1.330
Crystal dimensions [mm]	0.30 × 0.15 × 0.03	0.25 × 0.20 × 0.20
Radiation type	CuKα	CuKα
*μ* [mm^−1^]	1.080	0.853
		
**Data collection**		
Reflections measured	22714	33484
Range/indices (*h*, *k*, *l*)	−9,−9; −9, 9; −13, 13	−9, 9; −11, 10; −17, 17
θ (max, min) [°]	80.4, 4.2	80.3, 3.3
Total no. of unique data	2466	3697
No. of observed data, I > 2σ(I)	2239	3022
*R*_int_	0.042	0.043
		
**Refinement**		
*R* [*F*^2^ > 2σ (*F*^2^)]	0.035	0.058
*wR*(*F*^2^)	0.097	0.185
*S*	1.08	1.11
No. of reflections	2466	3697
No. of parameters	174	228
No. of restraints	0	0
H atom treatment	H atoms treated by constrained refinement	H atoms treated by constrained refinement
Δρ (min, max), e/Å^3^	−0.28, 0.27	−0.28, 0.35

**Table 3:** Energetical parameters for the HDA reaction between ethyl 4,β-dinitrocinnamate (DNC) (**1**) and methylenecyclopentane (**2**) in the toluene solution in light of the ωB97xd/6-311+G(d) (PCM) calculations.

Path	Transition	ΔH [kcal/mol]	ΔS [cal/molK]	ΔG [kcal/mol]	Imaginary Frequencies [cm^−1^]
	**1**+**2→MCA**	−6.5	−36.0	4.2	
**A**	**1**+**2→TSA**	11.5	−49.7	26.4	−389.3
	**1**+**2→3**	−27.3	−50.4	−12.2	
	**1**+**2→MCB**	−6.6	−36.1	4.2	
**B**	**1**+**2→TSB**	27.8	−49.0	42.4	−414.1
	**1**+**2→4**	−24.9	−53.0	−9.1	

**Table 4:** Key parameters for critical structures of the HDA reaction between ethyl 4,β-dinitrocinnamate (DNC) (**1**) and methylenecyclopentane (**2**) in the toluene solution in light of the ωB97xd/6-311+G(d) (PCM) calculations.

Path	Structure	Interatomic Distances [A]						GEDT
		O1-N2	N2-C3	C3-C4	C4-C5	C5-C6	C6-O1	[e]
	**1**	1.214	1.475	1.330				
	**2**					1.329		
**A**	**MCA**	1.212	1.475	1.330	3.629	1.330	4.282	0.00
	**TSA**	1.238	1.418	1.404	2.029	1.372	2.991	0.41
	**3**	1.397	1.317	1.502	1.536	1.526	1.479	
	**MCB**	1.213	1.474	1.330	3.700	1.326	3.649	0.00
**B**	**TSB**	1.258	1.386	1.423	1.973	1.381	2.366	0.40
	**4**	1.396	1.311	1.510	1.551	1.512	1.433	

**Table 5:**
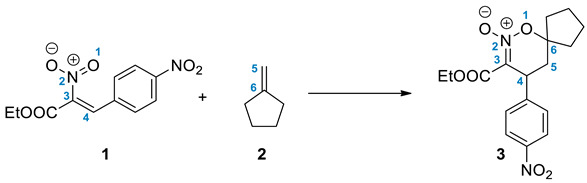
ELF valence basin populations and distances of the forming bonds based on ωB97xd/6-311+G(d) (PCM) calculations of the IRC structures, **MCA**–**3**, defining the ten different phases characterizing the molecular mechanism of the HDA reaction between ethyl 4,β-dinitrocinnamate (DNC) (**1**) and methylenecyclopentane (**2**). Distances are given in angstroms, Å, and electron populations in average number of electrons, e.

Points	1	2	MCA		P1		P2		P3		P4		P5		P6		P7		P8		P9		3
Phases				*I*		*II*		*III*		*IV*		*V*		*VI*		*VII*		*VIII*		*IX*		*X*	
d(C4-C5)			2.935		2.564		2.286		2.205		2.072		2.060		2.023		2.017		1.553		1.526		1.525
d(C6-O1)			3.316		3.133		3.044		3.025		2.999		2.996		2.989		2.988		2.103		1.585		1.499
V(O1,N2)	1.88		1.84		1.81		1.79		1.78		1.74		1.73		1.73		1.72		1.42		1.21		1.21
V(O1)	2.82		2.82		2.84		2.86		2.88		2.89		2.89		2.90		2.90		2.94		2.65		2.60
V’(O1)	2.77		2.82		2.84		2.85		2.84		2.85		2.85		2.86		2.86		2.92		2.39		2.37
V(N2,C3)	2.23		2.29		2.32		2.36		2.56		2.75		2.76		2.77		2.78		3.26		3.57		3.60
V(N2)	0.23		0.20		0.19		0.18																
V’(N2)	0.18																						
V(C3,C4)	1.80		1.84		3.47		3.47		3.47		2.96		2.86		2.64		2.62		2.04		2.04		2.03
V’(C3,C4)	1.74		1.66																				
V(C5,C6)		1.78	1.76		1.75		3.29		3.26		3.22		3.21		2.84		2.82		2.15		2.02		2.01
V’(C5,C6)		1.77	1.71		1.66																		
V(C3)											0.51		0.51		0.55		0.56		0.69		0.57		0.57
V(C4)													0.11		0.16								
V(C5)															0.37								
V(C4,C5)																	0.53		1.74		1.84		1.84
V(C6)																			0.06				
V(C6,O1)																					1.12		1.43

## Data Availability

The original contributions presented in this study are included in the article. Further inquiries can be directed to the corresponding author.
